# Panels of chemically-modified heparin polysaccharides and natural heparan sulfate saccharides both exhibit differences in binding to Slit and Robo, as well as variation between protein binding and cellular activity†
†Electronic supplementary information (ESI) available: NMR chemical shift characterisation of modified heparins, protein sequence alignment methodology and data, protein binding and activity assay dose-response curves. See DOI: 10.1039/c6mb00432f



**DOI:** 10.1039/c6mb00432f

**Published:** 2016-08-04

**Authors:** Yassir A. Ahmed, Edwin A. Yates, Diana J. Moss, Markus A. Loeven, Sadaf-Ahmahni Hussain, Erhard Hohenester, Jeremy E. Turnbull, Andrew K. Powell

**Affiliations:** a Centre for Glycobiology , Institute of Integrative Biology , University of Liverpool , UK; b Department of Chemistry , Faculty of Science , King Faisal University , Kingdom of Saudi Arabia; c Department of Cellular and Molecular Physiology , University of Liverpool , UK; d Department of Life Sciences , Imperial College London , UK; e School of Pharmacy and Biomolecular Sciences , Liverpool John Moores University , Liverpool , UK . Email: A.Powell@ljmu.ac.uk

## Abstract

Heparin/heparan sulfate (HS) glycosaminoglycans are required for Slit–Robo cellular responses. Evidence exists for interactions between each combination of Slit, Robo and heparin/HS and for formation of a ternary complex. Heparin/HS are complex mixtures displaying extensive structural diversity. The relevance of this diversity has been studied to a limited extent using a few select chemically-modified heparins as models of HS diversity. Here we extend these studies by parallel screening of structurally diverse panels of eight chemically-modified heparin polysaccharides and numerous natural HS oligosaccharide chromatographic fractions for binding to both *Drosophila* Slit and Robo N-terminal domains and for activation of a chick retina axon response to the Slit fragment. Both the polysaccharides and oligosaccharide fractions displayed variability in binding and cellular activity that could not be attributed solely to increasing sulfation, extending evidence for the importance of structural diversity to natural HS as well as model modified heparins. They also displayed differences in their interactions with Slit compared to Robo, with Robo preferring compounds with higher sulfation. Furthermore, the patterns of cellular activity across compounds were different to those for binding to each protein, suggesting that biological outcomes are selectively determined in a subtle manner that does not simply reflect the sum of the separate interactions of heparin/HS with Slit and Robo.

## Abstract 2

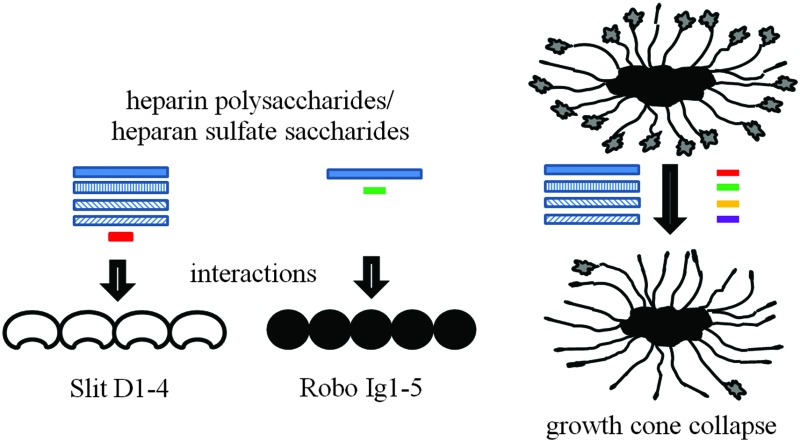
Panels of heparin/heparan sulfate carbohydrates differ in their interactions and bioactivity with Slit and Robo.

## Introduction

Slits are a family of secreted, multidomain glycoproteins which are ligands for the transmembrane immunoglobulin (Ig) superfamily proteins called roundabouts (Robos).^1,2^ Slit–Robo signalling was originally identified as being important in axon guidance^3,4^ but has since been found to have numerous biological roles^5–7^ and some association with human pathologies.^8^

Several experiments have suggested that cellular Slit activity requires heparan sulfate proteoglycans (HSPGs) as a co-receptor. In particular, *ex vivo* studies showed that human (h) Slit2 activity on rodent forebrain explants or *Xenopus* retinal axon growth cones can be eliminated by enzymatic degradation of cell-surface heparan sulfate (HS) chains.^9–11^ In addition, through genetic studies, Slit and Robo have also been associated with enzymes involved in HS biosynthesis, or the core proteins of HSPGs in *C. elegans, Drosophila* and mice.^12–17^ HS or heparin (a natural, highly sulfated variant of HS which is more readily-available, pharmaceutically important and often used as a proxy for HS) interact with both Slit and Robo.^10,18–21^ Heparin also affects binding of Slit to Robo which suggests formation of a ternary complex.^10^

**Fig. 1:**
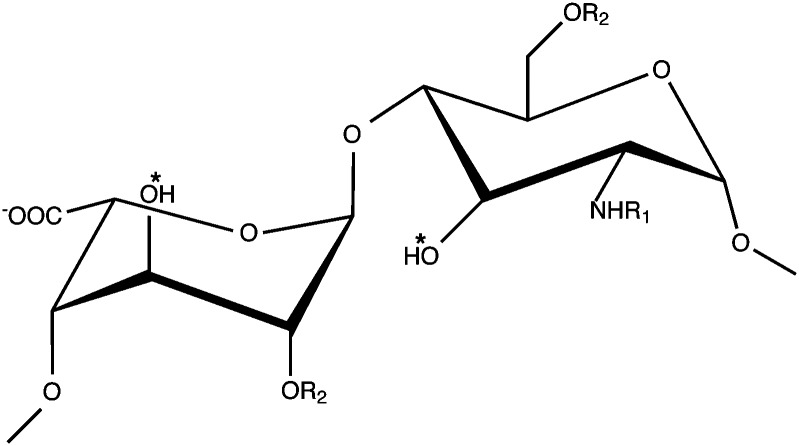
Schematic of the structure of heparin/HS disaccharide repeats. Depicted are α-l-iduronate and α-d-glucosamine. R_1_ = H, acetyl or SO_3_^–^ and R_2_ = H or SO_3_^–^. α-l-iduronic acid is formed from β-d-glucuronic acid through epimerization of the carboxyl group. * indicates sites where there is a major increase in sulfation during oversulfation of heparin.

HS and heparin are highly heterogeneous glycosaminoglycans (GAGs) that possess a repeating disaccharide unit consisting of uronic acid (β-d-glucuronic acid or α-l-iduronic acid) and α-d-glucosamine. The uronic acid can possess a hydroxyl or sulfate group at position-2, whilst glucosamine can exhibit either group at position-6 and position-3. Glucosamine can also display an *N*-acetyl, *N*-sulfonamido or free amino group at position-2 (Fig. 1).

This structural variation in disaccharide sulfation and epimerisation has been shown to influence protein binding and regulation of protein activity.^22^ However, only limited information exists regarding the effect of this diversity on the Slit/Robo system. Such an effect has been investigated for a few protein systems using chemically-modified heparin polysaccharides and (less commonly) chromatographic fractions of tissue-derived HS oligosaccharides which display variable sulfation.^23,24^ To date, Slit and Robo interactions have been studied separately and using only a few chemically-modified heparin polysaccharides that exhibit very limited diversity.^19,20,25^ Here we investigate the implications of HS structural diversity for the Slit/Robo system using a broad panel of eight structurally-diverse chemically-modified heparins, as model polysaccharides, in combination with a panel of numerous saccharide chromatographic fractions generated from tissue HS. This enables us to assess the consequences of diversity within natural HS as well as chemically-modified heparin model compounds and also to assess both polysaccharides and oligosaccharides that may differ in physical properties and biochemical activities.^21,23^ We screened their abilities to interact with N-terminal fragments of both *Drosophila* (d) Slit and dRobo through using parallel protein binding assays, enabling direct protein comparison for the first time. Furthermore, using an *ex vivo* chick retinal axon collapse assay, we screened their ability to promote the cellular activity of the dSlit fragment. The data suggest structural selectivity and clear differences between the profiles for binding to the different proteins and for protein binding and promotion of cellular activity.

## Results and discussion

### dRobo Ig1–5 and dSlit D1–4 exhibit subtly different interactions with chemically-modified heparin polysaccharides

**Fig. 2:**
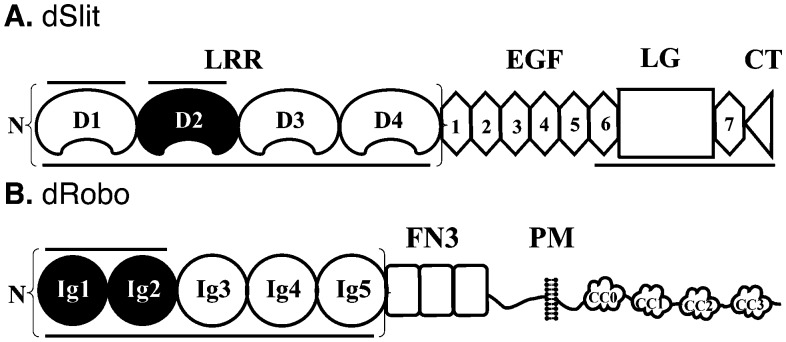
Schematic showing the domain structure of dSlit and dRobo. (A) dSlit showing D1–4 leucine-rich repeat (LRR), epidermal growth factor-like (EGF) and laminin G-like (LG) domains along with the C-terminal cystine-knot (CT). (B) dRobo showing immunoglobulin-like (Ig1–5), fibronectin type 3-like (FN3); transmembrane (TM) and conserved cytosolic motifs (CC0–3) domains. N represents N-terminus. Black colour filling represents dRobo and dSlit binding domains. Horizontal lines show HS-binding fragments. Brackets show protein fragments used in this study.

The importance of structural diversity within heparin/HS mixtures for the Slit/Robo system has only been investigated previously in separate studies, using a few select chemically-modified heparins of limited diversity with either hSlit proteins^19,25^ or a short hRobo1 fragment.^20^ To compare the effect of diversity on binding to Slit and Robo in parallel, we investigated *in vitro* binding of a broad panel of eight chemically-modified heparins to both dSlit and dRobo. Chemical modification involves the introduction of systemic variation in sulfation pattern compared with the unmodified parental heparin (Table 1 and Fig. 1). The resulting variants are structurally well-defined (ESI† Page S3) and known to exhibit distinct conformations and activities.^26^ We used proteins from *Drosophila* which expresses only a single Slit. DSlit has N-terminal and C-terminal heparin binding sites (Fig. 2), with N-terminal LRR domains having been shown to be sufficient for Robo binding and bioactivity.^10^ For simplicity, only the leucine-rich repeat (LRR) domains of dSlit were used. LRR domains D1–D4 were selected (Fig. 2) because, although D1 and D2 alone have been shown to bind to heparin (and D2 to bind Robo and exhibit bioactivity), D1–4 has a higher apparent affinity for heparin.^10^ *Drosophila* expresses three Robos and we used dRobo. Similar to dSlit, the N-terminal (Ig) domains of dRobo are sufficient for dSlit or heparin binding (Fig. 2). Again, although only dRobo Ig1–2 was found to be required for interactions with heparin or Slit, Ig1–5 was used (Fig. 2) as it has a slightly higher apparent affinity for heparin.^10,27^

**Table 1:** Characteristics of chemically-modified heparin compounds. I and A represent iduronate and glucosamine, respectively. 2S, 2OH, 6S, 6OH, NS, NAc represent sulfate (S), hydroxyl (OH) and acetyl substitutions at positions 2, 6 and *N* of iduronate or glucosamine

Compound	Predominant disaccharide repeat	Short-hand	Average sulfation per disaccharide
1	I_2S_A^6S^_NS_	Heparin	3
2	I_2OH_A^6S^_NS_	2-OH	2
3	I_2S_A^6OH^_NS_	6-OH	2
4	I_2S_A^6S^_NAc_	N-Ac	2
5	I_2OH_A^6S^_NAc_	2-OH/N-Ac	1
6	I_2S_A^6OH^_NAc_	6-OH/N-Ac	1
7	I_2OH_A^6OH^_NS_	2-OH/6-OH	1
8	I_2OH_A^6OH^_NAc_	2-OH/6-OH/N-Ac	0
9	I_2S,3S_A^6S^_3S,NS_	Oversulfated (OS)	5

**Fig. 3:**
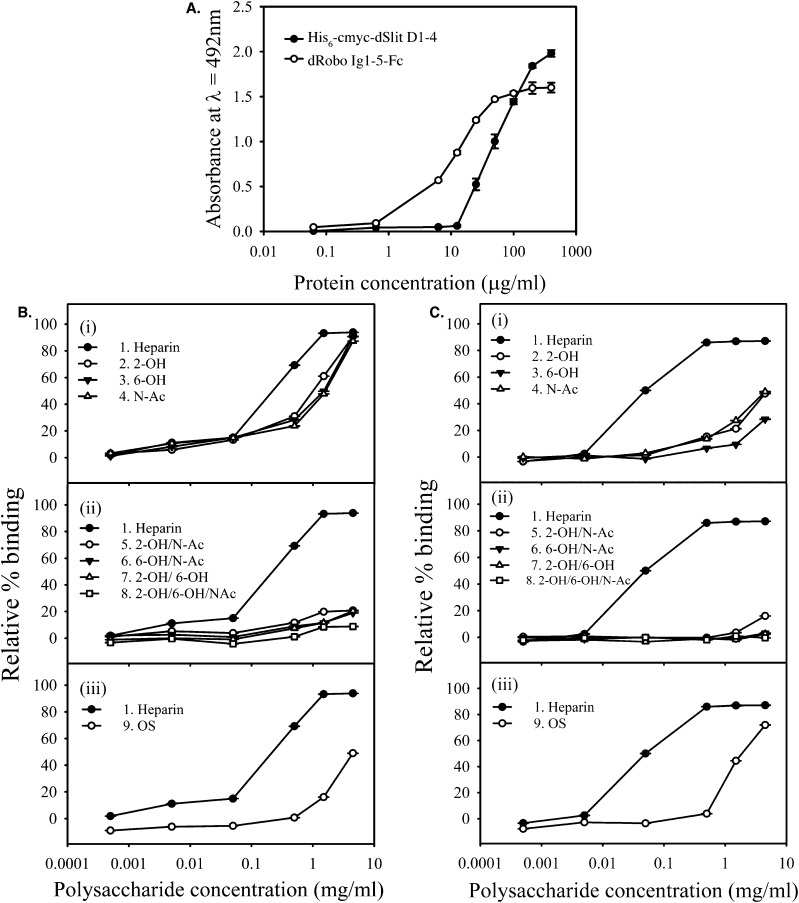
dSlit D1–4 and dRobo Ig1–5-Fc interact differently with heparin and chemically-modified heparins. (A) Binding of different concentrations of his_6_-cmyc-dSlit D1–4 and dRobo Ig1–5-Fc to a nitrous acid generated heparin 10-mer SEC fraction, surface-immobilised through biotinylation and streptavidin capture. Binding was determined using an ELISA and absorbance values shown are the mean of triplicates and error bars represent the standard deviation. Data are representative of three separate experiments. Binding of (B) his_6_-cmyc-dSlit D1–4 and (C) dRobo Ig1–5-IgG1Fc fusion protein to varying concentrations of soluble chemically-modified heparins (Table 1) determined by inhibition of protein binding to an immobilized ∼10-mer heparin saccharide fraction using a competition ELISA. (i) Compound 1 (heparin), 2 (2-OH), 3 (6-OH), 4 (N-Ac), (ii) compound 1 (heparin), 5 (2-OH/N-Ac), 6 (6-OH/N-Ac), 7 (2-OH/6-OH), 8 (2-OH/6-OH/N-Ac) and (iii) compound 1 (heparin) and 9 (OS). % binding values shown represent means of triplicate wells containing competitor relative to means of triplicate wells lacking competitor and error bars represent the % combined standard deviation. All calculations were performed as described in Experimental procedures. Data are representative of four separate experiments.

Dose–response experiments showed that dSlit D1–4 required higher concentrations compared with dRobo Ig1–5-Fc for binding to immobilised heparin oligosaccharides from a size exclusion chromatography (SEC) fraction (Fig. 3A), suggesting differential protein binding, with dSlit D1–4 binding being weaker. This was confirmed using a competition ELISA (where binding of polysaccharides is assessed through their ability to compete with the surface immobilised saccharide^28^), in which parental unmodified heparin (compound 1) was found to bind dSlit D1–4 less strongly (*i.e.* requiring higher concentrations of heparin: EC_50_ ∼ 7-fold higher) than dRobo Ig1–5-Fc (Fig. 3Bi and Ci).

Furthermore, heparins modified at a single position within disaccharide repeats (compounds 2–4) showed lower binding compared to parental heparin, but the reduction in binding was less pronounced for dSlit D1–4 than for dRobo Ig1–5-Fc (Fig. 3Bi and Ci). Heparins desulfated at two or three positions within disaccharide repeats (compounds 5–8) uniformly showed relatively very poor binding to both dSlit D1–4 and dRobo Ig1–5-Fc (Fig. 3Bii and Cii). Finally, oversulfated (OS) heparin (compound 9) possessing increased sulfation at two additional positions within the disaccharide repeats to parental unmodified heparin (see Table 1 and Fig. 1), also displayed reduced binding to dSlit D1–4 and dRobo Ig1–5-Fc compared with unmodified heparin, which was to a slightly greater extent for the dRobo fragment (Fig. 3Biii and Ciii).

Overall, these data suggest that binding of heparin and chemically-modified heparins to dSlit and dRobo N-terminal domains differs slightly: dRobo domains bind more strongly than dSlit to heparin and this binding is affected more by both desulfation and oversulfation of heparin. The reduction of polysaccharide binding to the proteins by oversulfation, as well as desulfation, of heparin suggests a degree of structural selectivity involving a particular range of sulfation or factors other than the extent of sulfation. Although it is possible that the difference in binding between his_6_-cmyc-dSlit D1–4 and dRobo Ig1–5-Fc reflect the Fc moiety causing dimerization of dRobo Ig1–5-Fc, which in turn results in an avidity effect on binding to the heparin variants, it is likely that the receptor domains can act independently as part of a dimeric Fc fusion protein, hence avidity effects may not occur.^28^

Comparison of our new data on binding of Slit with that of previous studies indicates subtle differences. We observed that binding of dSlit D1–4 to heparin was reduced almost equally by alteration at either of the 2, 6, or *N*-positions. In contrast, using only two variants (*N*-desulfated and *N*,*O*-desulfated heparins that are likely to possess positive charges on free amino groups at pH 7.4 of the binding assays), others found that O-sulfate, but not *N*-sulfate groups are required for modified heparins to compete with the interaction of rat glypican-1-Fc with full length hSlit2.^19^ Changes made selectively at the *N*-position in our work involved *N*-desulfation/re-*N*-acetylation hence the compounds do not possess similar modification, thus properties, at the *N*-position. Alternatively, using four variants, including one with *N*-modification similar to one of our variants, Shipp and Hsieh-Wilson^25^ observed that N-, 6-O- and 2-O-modified heparins bound to full length hSlit2 in decreasing order, with the latter exhibiting negligible binding. Our data suggest less difference between the binding abilities of these variants. This subtle discrepancy may reflect the use of different Slit proteins and binding assays. The full length hSlit2 protein used by Shipp and Hsieh-Wilson has two binding sites for heparin of different apparent affinities, with the C-terminal domain having a higher apparent affinity than the D1–4 region. Furthermore, Shipp and Hsieh-Wilson used glycan array methodology with limited heparin spotting concentrations (50, 25 and 15 μM) and which also depends on passive immobilisation of the polysaccharides on the array surface that may alter the apparent affinities of variants.^25^ In contrast, our study used a dSlit protein fragment containing only the N-terminal LRR domains (which have been shown to be sufficient for biological activity) and a competition assay with a wider range of chemically-modified heparin structures across a wide range of concentrations, equivalent to ∼80 nM to 300 μM.

In terms of Robo, our observation in the present study that single modification at the different major positions within disaccharide repeats substantially reduced dRobo Ig1–5-Fc binding (with loss of 6-O sulfates having the largest effect) is in agreement with previous work using hRobo1 Ig1–2.^20^ However, comparison is again made difficult by the use of different competition assays, proteins and heparin variants (*e.g. N*-desulfated rather than *N*-desulfated/re-*N*-acetylated). Overall, this comparison of studies demonstrates the importance of using parallel studies, that involve the same assay format and carbohydrate compounds, when comparing the structural features in heparin that are involved in binding to both Slit and Robo.

### Chemically-modified heparin polysaccharides differentially regulate dSlit D1–4 cellular activity.

Previously, the activities of full length or fragments of hSlit2 have been assessed using mouse, rat, frog and chick *ex vivo* explant assays.^9–11,29,30^ To our knowledge, no *ex vivo* Slit activity assay exists for *Drosophila* to match the dRobo used in the binding assays. Therefore, to assess the effect of diversity in regulation of dSlit D1–4 cellular activity, we developed a convenient *ex vivo* assay employing axons from chick retina,^31^ which is similar to the assay used previously with hSlit2.^29,30^ There is a single Slit gene in invertebrates and three in vertebrates.^2^ In contrast, there are three Robo genes in *Drosophila*, *Xenopu*s and chick and four in mammals.^32,33^ Slits from different species have 41–44% homology.^2^ DSlit or a dSlit D2 fragment have been shown to bind both rat Robo1 and 2 and to induce a human cell response, respectively, suggesting conservation of function across vertebrates and invertebrates.^2,27^ It is known that retina from chick embryos express Robo1 and Robo2.^29^ Sequence alignment showed 44 and 48% identity between Ig1–5 domains of dRobo and chick (c)Robo1 and 2, respectively (ESI,† Pages S4 and S5). However, it has been shown previously that Robo Ig1 possesses the key determinants for binding to Slit and heparin.^10,27,34^ Higher identities were calculated between Ig1 of dRobo with Ig1 of cRobo1 (58%) and 2 (57%).

**Fig. 4:**
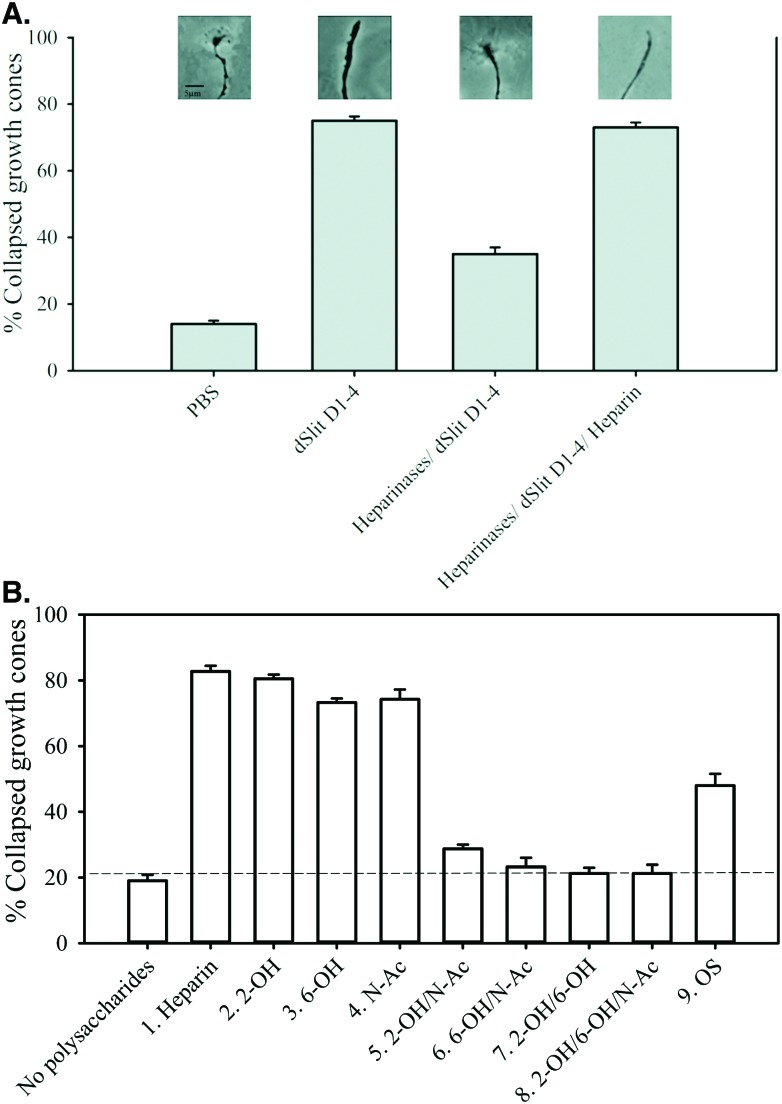
Chemically-modified heparins divide into groups with high, medium or low abilities to support dSlit D1–4 cellular activity. Slices of chick retina were treated *ex vivo* with (A) PBS, his_6_-cmyc-dSlit D1–4, heparinase I, II and III then his_6_-cmyc-dSlit D1–4 and heparinase I, II and III then his_6_-cmyc-dSlit D1–4 with exogenous heparin, (B) heparinase I, II, III then his_6_-cmyc**-**dSlit D1–4 in the presence or absence of chemically-modified heparins (Table 1): compound 1 (heparin), 2 (2-OH), 3 (6-OH), 4 (N-Ac), 5 (2-OH/N-Ac), 6 (6-OH/N-Ac), 7 (2-OH/6-OH), 8 (2-OH/6-OH/N-Ac) and 9 (OS). Collapsed and uncollapsed growth cones were counted in blind conditions across several pieces of retina for ∼100 growth cones and the % of collapsed growth cones calculated. Values shown are the mean % of collapsed growth cones calculated from three groups of retinal pieces and error bars represent the standard deviation for % values. Data are representative of two separate experiments.

Addition of dSlit D1–4 in PBS to retinal axons caused ∼77% collapse of growth cones compared to ∼15% for PBS alone indicating that the protein was biologically active (Fig. 4A). Removal of endogenous HS from the axons using heparinases substantially reduced the growth cone collapse activity of dSlit D1–4 (Fig. 4A). Addition of the protein in the presence of exogenous heparin fully restored activity on heparinase-treated growth cones (Fig. 4A). Having established that removal of endogenous HS from chick retinal axons using heparinases prevented dSlit D1–4 activity, which could be rescued by addition of exogenous heparin, we determined the relative rescue activities of chemically-modified heparins. Addition of dSlit D1–4 alongside heparins modified at single positions within the disaccharide repeats (compounds 2–4), restored growth cone collapse activity to similar level (∼75–80%) to parental unmodified heparin (compound 1: ∼82%), thus placing these variants with unmodified heparin in a high activity group (Fig. 4B). In contrast, heparins modified at two or three positions within their disaccharide repeats (compounds 5–8), exhibited a much lower level of rescue (∼20–30% collapse), thus placing these variants in a low activity group (Fig. 4B). This lower activity was similar to that of dSlit D1–4 without addition of any polysaccharide (∼20% collapse) (Fig. 4B). OS heparin (compound 9) fell between the high and low activity groups (∼50% collapse) clearly indicating that additional sulfation is not optimal for promoting activity (Fig. 4B).

Overall the data demonstrate that sulfation levels in these relatively homogenously modified heparins regulate their abilities to control the biological response of chick retinal axons to dSlit D1–4. Similar to protein binding, the reduction of polysaccharide activity by oversulfation, as well as desulfation, of heparin suggests a degree of structural selectivity involving a particular range of sulfation or factors other than the extent of sulfation. Addition or removal of sulfates at two or more positions in the regular disaccharide repeats of modified heparins had a substantial deleterious effect similar to that observed on dSlit D1–4 or dRobo Ig1–5-Fc binding. Modification of heparin at a single position within disaccharide repeats, however, had little effect on cellular activity, in contrast to larger effects observed on binding to the individual proteins.

As observed for protein binding, our results are not entirely in agreement with previous observations. Firstly, Shipp and Hsieh-Wilson demonstrated varied effects of modification at a single position within the disaccharide repeats of heparin on hSlit2 cellular activity.^25^ In this case, the difference in results may be due to use of different cellular assays or Slit proteins. Others have also reported effects of modification on *in vivo* activities suspected to be linked with Slit–Robo signalling. Firstly, 2- and 6-O desulfation and to a lesser extent *N*-desulfation/*N*-re-acetylation were found to affect *in vivo Xenopus* axon guidance.^35^ Secondly, HS 2-O-sulfotransferase and HS 6-O-sulfotransferase-1 knockout mice (exhibiting either altered 2-O or 6-O sulfation) have altered axon guidance phenotypes shown to be associated with Slit function.^16,36^ However for these more complex *in vivo* assays other proteins may also be involved preventing direct comparison with our *ex vivo* Slit cellular activity assay. Collectively, this suggests the need for caution to avoid over-simplistic interpretation or comparison of different binding or activity datasets as the experimental context influences the detail of the results.

### Generation of structurally diverse HS saccharide chromatographic fractions

**Fig. 5:**
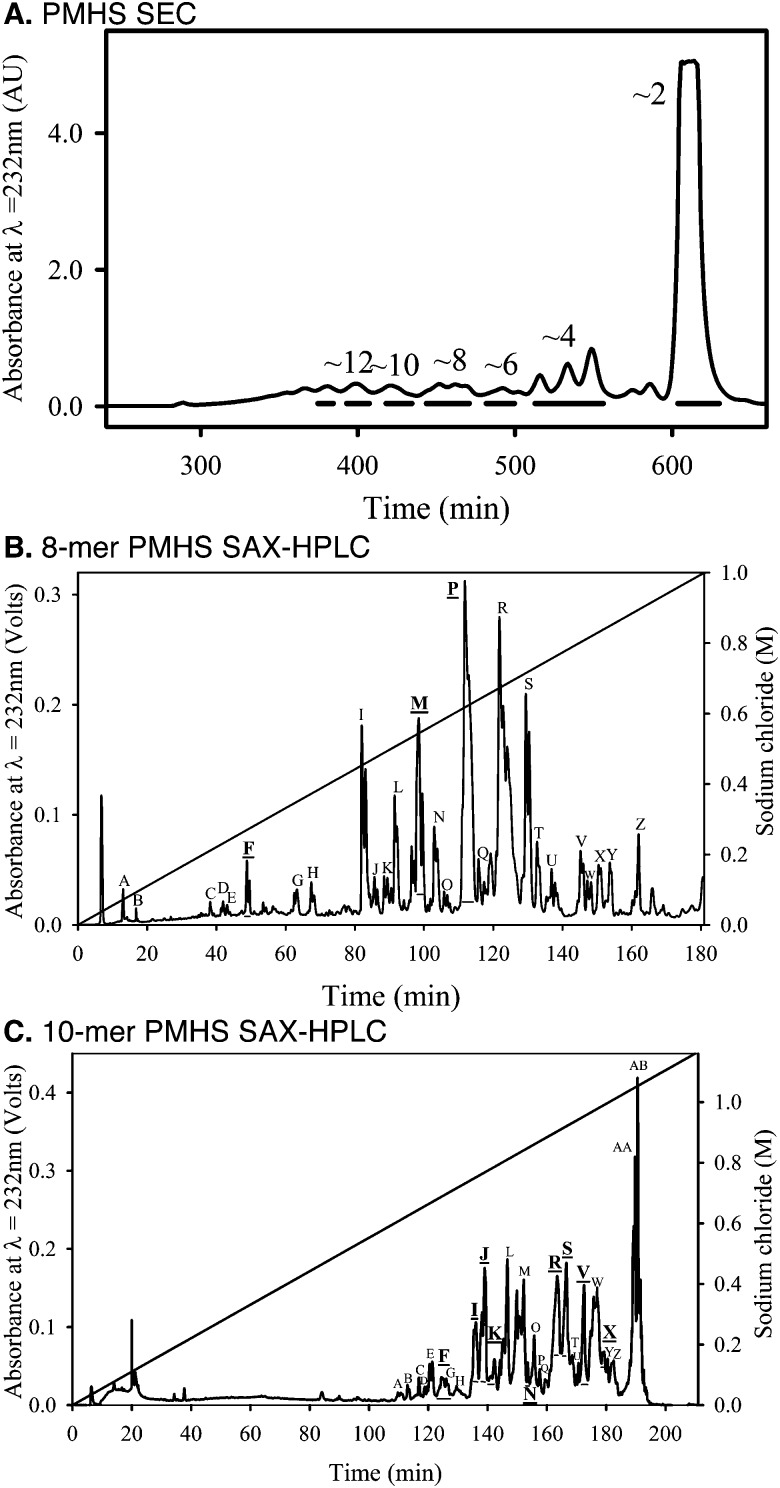
Generation of saccharide chromatographic fractions. PMHS was partially digested with heparinase III and saccharide mixtures separated by (A) SEC. Fractions from SEC that contain (B) ∼8-mer and (C) ∼10-mer saccharides were then further separated by SAX-HPLC. Fractions across selected peaks were broadly estimated to contain HS/heparin oligosaccharides displaying increasing degrees of polymerization (shown by even integers). Pooling is shown by horizontal lines beneath peaks. SAX sub-fractions in underlined bold correspond to those used in assays. Chromatography elution profiles were reproducible across multiple runs.

In order to explore the effect of structural diversity within natural HS on the Slit–Robo system, we next investigated protein binding and activity using tissue-derived HS. As HS chains exhibit extensive structural diversity and are potentially multi-dentate for protein binding,^23^ we generated libraries of oligosaccharides by chromatographic separation of saccharide mixtures produced by partial heparinase III digestion of porcine mucosal HS (PMHS).^24^ It was shown previously that a ∼10-mer heparin saccharide fraction, generated by sequential size exclusion chromatography (SEC) and strong anion exchange (SAX)-HPLC, was able to form a ternary complex with dSlit D2 and dRobo Ig1–2.^10^ We therefore focussed on generating ∼8-mer and ∼10-mer fractions from PMHS.^24^ SEC was used to separate saccharides based upon hydrodynamic volume (Fig. 5A), which equates approximately to differences in saccharide length. PMHS ∼8-mer and ∼10-mer fractions were further separated based upon sulfation using SAX-HPLC at pH ∼ 3 (Fig. 5B and C). Fractions eluting from the SAX column at different salt concentrations mainly reflect saccharides possessing differential sulfation,^24^ hence a range of major saccharide fractions were selected across the gradients to screen for protein binding and cellular activity (Fig. 5B and C).

### PMHS saccharide fractions possess differential protein binding and cellular activity

**Fig. 6:**
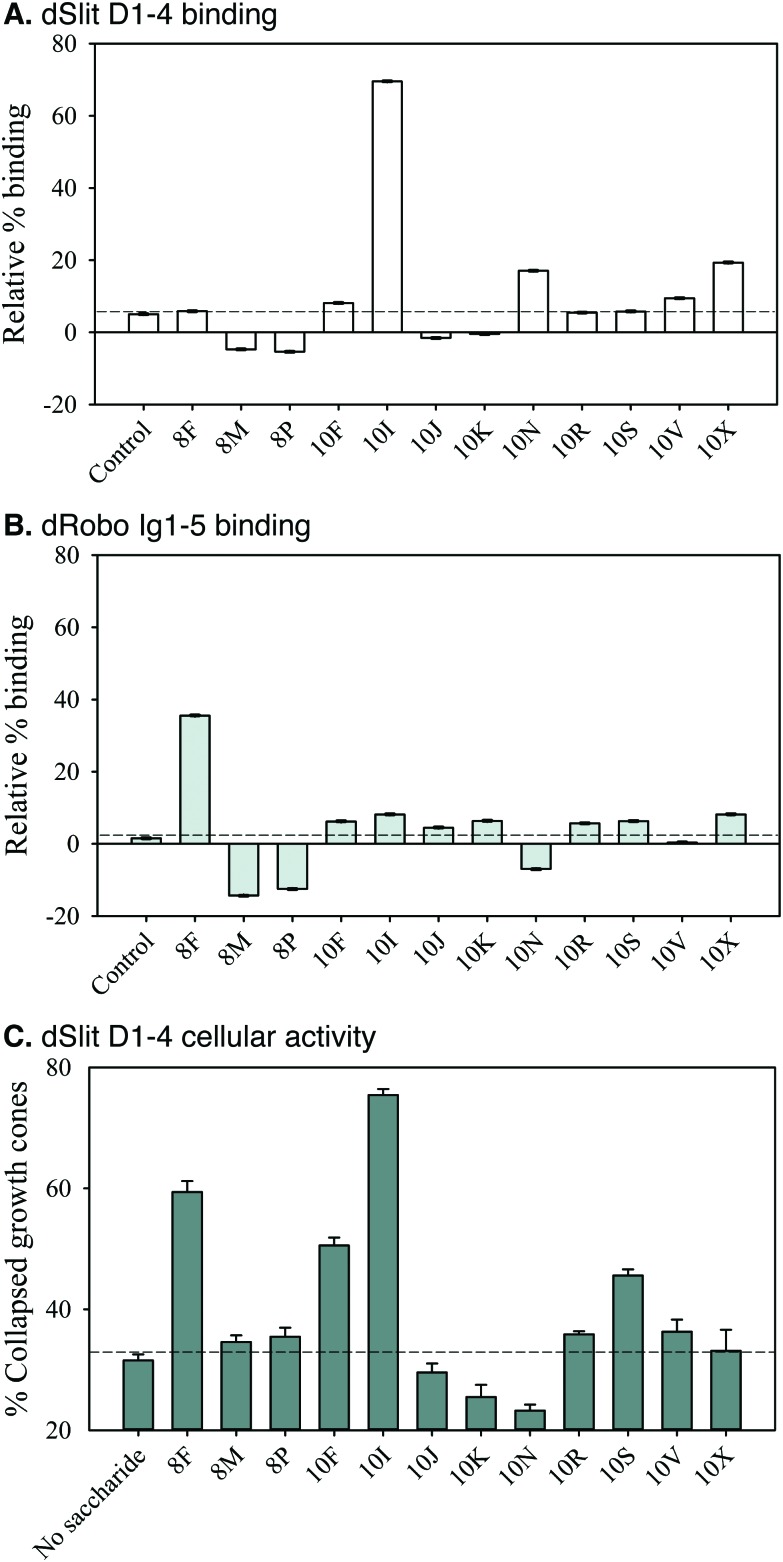
PMHS saccharides possess differential abilities to bind dSlit D1–4 or dRobo Ig1–5-Fc and promote dSlit D1–4 cellular activity. PMHS ∼8-mer and ∼10-mer SAX-HPLC fractions were screened for binding to (A) his_6_-cmyc-dSlit D1–4 and (B) dRobo Ig1–5-IgG1Fc fusion protein using a competition ELISA. % binding values shown represent means of triplicate wells containing competitor relative to means of triplicate wells lacking competitor and error bars represent the % combined standard deviation calculated as described in Experimental procedures. Control represents protein binding to a surface lacking immobilised biotinylated heparin saccharides. Data are representative of four separate experiments. Fractions were also screened for their ability to rescue (C) His_6_-cmyc-dSlit D1–4 *ex vivo* chick retinal axon growth cone collapse following treatment with heparinases I–III. Collapsed and uncollapsed growth cones were counted in blind conditions across several pieces of retina for ∼100 growth cones and the % of collapsed growth cones calculated. Values shown are the mean % of collapsed growth cones calculated from three groups of retinal pieces and error bars represent the standard deviation for % values. Data are representative of two separate experiments.

Screening of SAX-HPLC saccharide fractions in the competition ELISA for binding to dSlit D1–4 and dRobo Ig1–5-Fc indicated they possessed differential abilities (Fig. 6A and B). Interestingly, fraction 10I, which elutes at a relatively low salt concentration (∼0.75 M, Fig. 5C) indicating moderate sulfation, had a uniquely strong ability to bind dSlit D1–4 (∼70% relative binding, Fig. 6A). In marked contrast to dSlit D1–4, dRobo Ig1–5-Fc bound best to fraction 8F (∼35% relative binding, Fig. 6B). This fraction is particularly interesting as it eluted from the SAX column at the lowest relative salt concentration (∼0.3 M) (Fig. 5B) indicating low sulfation. All the other fractions exhibited low relative binding (<20%: Fig. 6A and B).

We next investigated the ability of fractions to promote dSlit D1–4 chick growth cone collapse activity. Similar to protein binding, the fractions had differential bioactivities (Fig. 6C). Again only a few had strong activities whilst others were similar to the residual 30% collapse exhibited in the absence of saccharides as a control. Fractions 10I and 8F, which had also been found to bind well to dSlit D1–4 or dRobo Ig1–5, respectively (Fig. 6A and B), exhibited the highest (∼75%) and next highest (∼60%) activities (Fig. 6C). Interestingly, fractions 10F and 10S, which did not bind strongly to either dSlit D1–4 or dRobo Ig1–5-Fc (Fig. 6A and B) had medium growth cone collapse activity (∼50 and ∼45%, Fig. 6C). Despite possessing similar activities, these latter fractions also elute from the SAX column at different salt concentrations (0.7 M and 0.9 M, Fig. 5C) suggesting a different extent of sulfation.

Together the data suggest that binding of natural HS saccharides to both Slit and Robo fragments and their ability to stimulate a Slit-dependent biological response is not simply dependent on the degree of sulfation (as indicated by the concentration of NaCl for elution). Differential binding was observed for the two proteins. Furthermore, it appears that the relationship between binding and activity is again quite complex, with a couple of biologically active saccharide fractions (10F and 10S) binding poorly, at best, to either of the proteins and two other active saccharide fractions (10I and 8F) binding strongly to only one or other of the proteins.

## Conclusion

Previously, Slit and Robo interactions have been studied separately using only a few chemically-modified heparin polysaccharides exhibiting limited structural diversity.^19,20,25^ As it is difficult to compare across studies, the investigation of dSlit and dRobo binding by our study in parallel, using the same assays and compounds, enables direct comparison to be made for the first time. In our study Slit and Robo fragments displayed different binding characteristics. In general the dRobo fragment had a greater preference for binding to heparin than the dSlit fragment, as dRobo bound unmodified heparin more strongly and removal of sulfates from single positions reduced binding to a greater extent. Furthermore, the profiles for the binding of each protein to selected 8-mer and 10-mer PMHS fractions differed, especially as only a single different fraction bound strongly to either dSlit (10I) or dRobo (8F). Together these observations suggest that the ligand and receptor exhibit subtle differences in their preferences for structural characteristics of partner HS saccharides. This subtle difference in ligand and receptor binding could be significant, as in the case of the fibroblast growth factor (FGF) system where FGF ligand and FGF receptor binding to heparin/HS have been shown to be distinct.^37^ Furthermore, as there are different isoforms of Robo, selectivity for binding to Robo could be important, as is the case for the FGF system in which different HS/receptor interactions occur^28^ and affect the output of FGF signalling.^38^

Comparing chemically-modified heparins across individual protein binding and cellular activity assays suggests that removing sulfates from two or more positions within the disaccharide repeats has a substantial effect. The data for removal of sulfates from a single position within disaccharide repeats are more complex in that they show a reasonable influence on protein binding but not on growth cone collapse activity. Similarly, differences are seen between HS saccharide profiles for protein binding compared with cellular activity. These indicate that there is no simple direct correlative relationship between protein binding and cellular activity. This probably reflects the requirement for formation of a ternary complex^10^ with a particular architecture, which binding to a single ligand or receptor protein alone does not resemble. Similar observations have again been made for the FGF system.^39,40^

Overall, our results suggest that structural diversity within HS GAGs affects the Slit–Robo system thus there is clearly potential for biological regulation of the Slit–Robo system *via* variations in HS structure. This proposal is supported by knockout studies in *C. elegans* and mice^12,16,36^ and is also observed for FGF signalling.^41^ Further studies (for example using a collection of pure, structurally-defined HS saccharides) should help to elucidate such mechanisms.

## Experimental

All common chemicals used were of analytical grade and purchased from VWR (Lutterworth, UK) unless otherwise stated. Porcine mucosal heparin (PMH) was purchased from Celsus Laboratories (Ohio, USA). Chemically-modified PMH polysaccharides were prepared and structurally characterised as formerly described.^42^ PMHS was a gift from Organon (Oss, Netherlands). All polysaccharides were quantified by weighing. Recombinant heparinases I, II and III from *Flavobacterium heparinum* were purchased from IBEX (Montreal, Canada); 1 IU is defined as the amount of enzyme that will liberate 1 μmol of unsaturated oligosaccharides from HS per minute at 30 °C and pH 7.5. His_6_-myc-tagged dSlit D1–4 and dRobo Ig1–5-IgG1Fc proteins were prepared and quantified, using absorbance at *λ* = 280 nm and molar extinction coefficients calculated from sequence, as previously described.^10^ Molecular weights were estimated from sequence as ∼103.5 and 80 KDa, respectively.

### Generation of PMHS saccharide chromotographic fractions for screening

PMHS saccharide fractions were generated using enzymatic cleavage and chromatographic procedures.^24^ PMHS (50 mg) was partially digested with 0.1 mU mg^–1^ (5 mU ml^–1^) of heparinase III for 2 h, adding a further 10 mU heparinase III and then again at 24 hours, before heating to 100 °C. Heparinase-generated saccharide mixtures were first separated by hydrodynamic volume using SEC with a Superdex™ 30 (GE Healthcare, UK) column (16 mm I.D. × 200 cm length) performed in 0.5 M ammonium hydrogen carbonate at a flow rate of 0.5 ml min^–1^. Peak fractions were collected and desalted using a HiPrep™ 26/10 Desalting column (GE Healthcare, UK) in water at 5 ml min^–1^ and concentrated using freeze drying. Selected SEC fractions were further separated by charge using high resolution, SAX-HPLC. For this procedure, a 9 × 250 mm Propac PA1 column (Dionex, UK) was equilibrated in double deionised water (pH ∼ 3.5) at a flow rate of 1 ml min^–1^. Sample (∼3 mg) was then loaded onto the column in water (∼pH 3.5) before elution with a linear gradient of sodium chloride (0–1 M, ∼pH 3.5 over 180 min), monitoring absorbance at *λ* = 232 nm for fraction collection. Multiple separations were performed and replicate fractions of peaks were pooled and desalted using the HiPrep™ 26/10 Desalting column run in water at 5 ml min^–1^ and then concentrated using freeze drying. SEC and desalting chromatography was performed using an AKTA purifier 10 (GE Healthcare), whilst SAX-HPLC used a Shimadzu HPLC with SCL-10A controller, LC-10AT pump, CS16150 vacuum degasser, FCV-10AL mixer, CTO-10 AS column oven, SPD-10 UV detector and Class-VP chromatography data system (Shimadzu, UK).

Saccharides in SEC fractions were quantified by weighting following lyophilisation as large amounts of saccharide were available (≥1 mg). The concentrations of saccharides in SAX-HPLC fractions were quantified using absorbance at *λ* = 232 nm and the molar extinction coefficient of 5500 mol^–1^ cm^–1^ for the unsaturated bond chromophore generated by heparinase enzymes.^43^ Molecular weights of different sized saccharides were estimated using 440 Da per disaccharide.

### Generation of biotinylated heparin ∼10-mer SEC fraction saccharides

PMH saccharide mixtures were generated using nitrous acid cleavage.^44^ Nitrous acid (pH ∼ 2.0) was freshly made by adding 0.5 M sodium nitrite to an equal volume of 0.5 M hydrochloric acid and the mixture kept on ice. An equal volume of the 250 mM nitrous acid solution was used to dilute 100 mg ml^–1^ heparin at room temperature before removing aliquots at 10, 20, 30 and 40 minutes and stopping the digestion by neutralisation with 1 M sodium hydrogen carbonate. Saccharide mixtures were then separated using the Superdex™ 30 SEC column as described above. For biotinylation, lyophilised ∼10-mer fraction was dissolved at 10 mg ml^–1^ in double deionised water (∼pH 5.0 using HCL). A five-fold molar excess of 2.5 mM aminoxybiotin [*N*-(aminooxyacetyl)-*N*′-(d-biotinoyl) hydrazine, trifluoroacetic acid salt (ARP)] (Invitrogen, UK) in double deionised water was added and the reaction mixture incubated for a minimum of 16 hours at 50 °C. Sequential runs over a 0.5 ml DEAE-Sephacel (GE Healthcare) column were used to remove free biotin reagent. The DEAE column was equilibrated with 50 mM NaCl/10 mM phosphate buffer (pH 7.0), the sample was loaded and washed with buffer to remove unreacted biotin (∼50 column volumes), before eluting bound oligosaccharides with 1 ml of 2 M NaCl. Absorbance of eluted fractions was monitored at *λ* = 232 nm using a spectrophotometer (Shimadzu, UK). Elution fractions were pooled and desalted using the HiPrep™ 26/10 Desalting column. Biotinylation of the ∼10-mer saccharide fraction was confirmed using a dot blot with a nitrocellulose membrane (Hybond-ECL, GE Healthcare, UK), blocking with 5% (w/v) BSA and probing with an anti-biotin-HRP fusion protein (Cell Signalling, UK).

### ELISAs

ELISAs were used to study HS-protein interactions.^28^ Streptavidin (3 μg ml^–1^; Promega, UK) in 0.1 M sodium carbonate/sodium hydrogen carbonate was incubated for 16 hours at 4 °C in Maxisorp 96-well microtiter plates (Nunc, UK). Plates were blocked with 1% (w/v) BSA in PBS for 2 hours at room temperature and washed with PBS, 0.05% (v/v) Tween 20 (Sigma, UK) (PBST), before incubation with the biotinylated PMH ∼10-mer SEC fraction saccharides in 1% (w/v) BSA/PBST for 2 hours at room temperature and further washing with PBST. For protein dose experiments, wells were incubated with varying concentrations of his_6_-myc-tagged dSlit D1–4 and dRobo Ig1–5-Fc in 1% BSA, PBST overnight at 4 °C. Alternatively, for competition ELISA experiments, fixed concentrations of dSlit D1–4 (60 μg ml^–1^: ∼55% of maximal binding to the immobilised PMH ∼10-mer: Fig. 3A) and dRobo Ig1–5-Fc (30 μg ml^–1^: ∼75% of maximal binding to the immobilised PMH ∼10-mer: Fig. 3A) were incubated under the same conditions with varying concentrations of polysaccharides. For oligosaccharide competition, the fixed concentrations of proteins detailed above were incubated with saccharide fractions, at concentrations that varied (for dose-determination using SEC fractions: ESI,† Page S6) or that were fixed (for screening of SAX fractions at 100 μg ml^–1^, corresponding to ∼50% or ∼25% competition of Slit and Robo binding by a PMH ∼10-mer SEC fraction, respectively: ESI,† Page S6). For dSlit D1–4 detection, mouse anti-myc (9E10) antibody (Sigma, UK) was added at 1 μg ml^–1^ and then sheep anti-mouse IgG-horse radish peroxidise (HRP) (GE Healthcare, UK) at 1 μg ml^–1^. dRobo Ig1–5-Fc detection used a goat anti-human IgG-HRP conjugate (Fisher Scientific, UK) at 0.2 μg ml^–1^. All antibodies were incubated in 1% BSA, PBST for 1 hour at room temperature. Antibody binding was detected using a solution of the HRP substrate (orthophenylenediamine) by following the manufacturer's instructions (Dako, UK) and measuring product absorbance at *λ* = 492 nm (A^492^) using a plate reader (Thermo, UK). A^492^ values were exported to Microsoft Excel 2010 and further calculations performed: to account for variance between plates in the HRP reaction time, relative% binding of proteins to polysaccharides or saccharide fractions were calculated using the formula: 100 – [(mean A^492^ in the presence of competitor/mean A^492^ in the absence of competitor) × 100]. Combined standard deviations for relative% binding were calculated using the formula SE = 100 – [Av_*x*_/Av_*y*_ √{(SD_*x*_^2^/Av_*x*_)^2^ + (SD_*y*_^2^/Av_*y*_)^2^} × 100], where *x* is A^492^ in the presence of competitor and *y* is A^492^ in the absence of competitor, Av is the mean value and SD the standard deviation. Graphs were generated using SigmaPlot V11 and, where appropriate, EC_50_s were calculated using non-linear regression with a 4-parameter logistic equation with the software, giving *R* ^2^ values of 0.99–1.

### Retinal axon growth cone collapse bioassay

Chick retina axon-growth-cone collapse assays were used to measure the activity of dSlit D1–4.^31^ For stage one, cover-slips (VWR, UK) were sterilized by oven heat and then coated with 70 μl 10 μg ml^–1^ sterile poly-l-lysine (Sigma, UK) in 100 mM sodium borate buffer pH 8.3 (Sigma, UK) for 1 hour. After incubation, the cover-slips were washed by dipping twice into HBSS medium (Invitrogen/Gibco, UK). Cover-slips were then coated with 30 μl of 10 μg ml^–1^ sterile laminin (Invitrogen, UK) in double deionised water and incubated for 1 h at room temperature. They were washed again by dipping into HBSS and placed into 4-well plates (Nunc/VWR, UK) with their coated side facing up. Each well was covered with 0.5 ml of retina culture medium [Hams F12 glutamax (Invitrogen/Gibco, UK), 0.5% (w/v) methylcellulose (Sigma, UK), 100 units per ml penicillin/streptomycin (Invitrogen/Gibco, UK), 5 μg ml^–1^ transferrin/insulin/selenium (Invitrogen/Gibco, UK), 100 μg ml^–1^ transferrin (Sigma, UK) and 100 μg ml^–1^ BSA (Sigma, UK)]. The 4-well plates were incubated at 37 °C until required.

The second stage was started by removing whole eyes from several E7 chick embryos. E7 chick embryos do not come under home office legislation and do not require ethical approval. These eyes were placed in Ham's F12 medium for 5 minutes at 37 °C which helps in the dissection step. All the subsequent steps in this stage were performed in Ham's medium and under sterile conditions. Dissection of each eye was started by removal of connective tissue (the whitish layer surrounding the eye), and followed by removal of the retinal pigmented epithelium (the dark brown or black layer). Finally, the lens was removed with the vitreous humor (gelatinous ball) attached. It was observed that retina began to curl after several minutes in medium. Retina extracted from the group of eyes, which were deemed suitable for use, were then cut into pieces of the required size (∼1 mm × 1 mm) using dissecting scissors. These pieces were then transferred to coverslips (3–4 pieces on each coverslip) and incubated in medium at 37 °C for 24 hours.

For the third stage, pieces of retina were checked for the growth of well-defined axon growth cones using an inverted microscope. Unsuitable pieces of retina were discarded. Pieces of retina were incubated at 37 °C for three hours in 500 μl of retina culture medium in the presence or absence of heparinases I, II and III (each added at 5 mU ml^–1^) as appropriate. This medium was then replaced with concentrations of dSlit D1–4 in PBS. To screen for the effect of GAGs, 10 μg ml^–1^ dSlit D1–4 (∼90% of maximal activity: ESI,† Page S7) was added in the presence or absence of polysaccharides and oligosaccharide fractions at concentrations that varied (dose identification: ESI,† Page S7) or that were fixed (200 μg ml^–1^ polysaccharide: ∼90% of maximum activity of PMH or 50 μg ml^–1^ oligosaccharide fraction: ∼65% of maximum activity of a ∼10-mer PMH SEC fraction, ESI,† Page S7). Solutions were incubated for 20 minute at 37 °C.

For the final stage, cover-slips were fixed by gently adding 250 μl of fixing medium (0.12 M sucrose, 0.5 mM CaCl_2_, 75 mM Millonig's Phosphate Buffer, 2% glutaraldehyde) (Sigma, UK) on one side of the well followed by incubation for 30 min at room temperature. Cover-slips were lifted up off the wells and washed twice with double deionised water, air dried at room temperature and mounted on a glass slide (two cover-slips per slide). PBS was found to be the best mounting solution (as many other commercially-available mounting solutions failed, due to the problem of air bubbles under the cover-slips making it difficult to properly assess the morphology of the growth cones). The cover slip was sealed around the edges using clear nail varnish. A Zeiss LSM 510 Meta microscope with LSM 510 software was used to count collapsed and uncollapsed growth cones across several pieces of retina until 80–110 axons had been assessed. Average and standard deviation values for % collapse across coverslips with replicate groups of slices from the retinal pool were calculated using Microsoft Excel 2010 and graphs generated using Sigma Plot V11.

## Author contributions

Y. A. A. performed the experiments and assisted A. K. P. analyse data. E. A. Y. generated chemically-modified heparins and D. M. developed the chick bioassay. S.-A. H. expressed and purified proteins. M. A. L. helped generate PMHS SAX-HPLC fractions. A. K. P. conceived and wrote the manuscript with assistance from Y. A. A., whilst E. A. Y., E. H. and J. E. T. provided comments on manuscript drafts. All authors approved the final version. A. K. P., E. H. and J. E. T. acquired supporting funding.

## Abbreviations

c: Chick
D: Domain
d: *Drosophila*
FGF: Fibroblast growth factor
GAG: Glycosaminoglycan
HS: Heparan sulfate
HSPG: Heparan sulfate proteoglycan
HRP: Horse radish peroxidise
h: Human
Ig: Immunoglobulin
OS: Oversulfated
PMH: Porcine mucosal heparin
PMHS: Porcine mucosal heparan sulfate
Robo: Roundabout
SEC: Size exclusion chromatography
SAX: Strong anion exchange
