# Supplementary material for: Panels of chemically-modified heparin polysaccharides and natural heparan sulfate saccharides both exhibit differences in binding to Slit and Robo, as well as variation between protein binding and cellular activity
Source: Mol Biosyst. 2016 Aug 4;12(10):3166–75. doi: 10.1039/c6mb00432f (PMC5048398; doi:10.1039/c6mb00432f)
Supplement: Supplementary file 1 [file MB-012-C6MB00432F-s001.pdf]

Electronic Supplementary Material (ESI) for Molecular BioSystems.
This journal is © The Royal Society of Chemistry 2016

Supporting Information for the manuscript:

Panels of chemically-modified heparin polysaccharides and natural heparan sulfate saccharides both exhibit differences in binding to Slit and Robo, as well as variation between protein binding and cellular activity.

Yassir A. Ahmed,[a,b] Edwin A. Yates,[a] Diana J. Moss,[c] Markus A. Loeven, [a] Sadaf-Ahmahni Hussain,[d] Erhard Hohenester,[d] Jeremy E. Turnbull, §[a] and Andrew K. Powell, §[a, e]

[a.] *Centre for Glycobiology, Institute of Integrative Biology, University of Liverpool, UK*
[b.] *Department of Chemistry, Faculty of Science, King Faisal University, KSA*
[c.] *Department of Cellular and Molecular Physiology, University of Liverpool, UK*
[d.] *Department of Life Sciences, Imperial College London, UK*
[e.] *School of Pharmacy and Biomolecular Sciences, Liverpool John Moores University, Liverpool, UK*

Contents:

Methodology. S2

Results. S3

References. S8

METHODOLOGY

Sequence alignment: Protein sequences were obtained from UniProt (http://www.uniprot.org) for dRobo (O44924), cRobo1 (F1NR30) and cRobo2 (A0A024B7I3). Amino acids corresponding to Ig1-5 (56-530, 29-502 and 29-506) and Ig1 (56-149, 29-125 and 29-125) for dRobo, cRobo1 and cRobo2, respectively were identified by entering UniProt IDs into the Prosite function in the ExPASy Bioinformatics Resource Portal (http://www.expasy.org/). Sequences were entered as pairs into the multiple sequence alignment programme PRALINE to generate alignments and calculate % identity (http://www.ibi.vu.nl/programs/PRALINEwww).

RESULTS

		Glucosamine (A)	Iduronate (I)
**Polysaccharide**	**Disaccharide Repeat**	**A-1 A-2 A-3 A-4 A-5 A-6**	**I-1 I-2 I-3 I-4 I-5**
**Heparin**	$I_{2S}A^{6S}{}_{NS}$	99.5 60.7 72.5 78.8 72.0 69.2	102.1 78.9 72.1 79.0 72.3
		5.42 3.31 3.69 3.79 4.05 4.30-4.42	5.23 4.37 4.22 4.14 4.82
**2-OH**	$I_{2OH}A^{6S}{}_{NS}$	98.1 60.3 72.4 80.1 71.5 68.7	104.6 71.1 70.4 77.2 71.2
		5.34 3.24 3.65 3.71 4.02 4.36 4.23	5.04 3.78 4.12 4.08 4.84
**6-OH**	$I_{2S}A^{6OH}{}_{NS}$	100.0 60.8 72.4 80.5 73.8 62.6	102.0 77.6 70.7 78.7 71.4
		5.31 3.27 3.71 3.70 3.89 3.86-3.88	5.26 4.35 4.25 4.06 4.84
**N-Ac**	$I_{2S}A^{6S}{}_{NAc}$	96.6 56.2 73.0 79.3 72.3 69.6	102.2 76.8 67.3 74.2 70.8
		5.15 4.03 3.76 3.78 4.04 4.31-4.37	5.20 4.37 4.31 4.08 4.91
**2-OH/N-Ac**	$I_{2OH}A^{6S}{}_{NAc}$	97.1 56.2 72.5 79.6 71.8 68.8	104.6 72.0 71.4 77.0 71.9
		5.18 4.00 3.78 3.79 4.08 4.37-4.26	5.01 3.75 3.42 4.10 4.78
**6-OH/N-Ac**	$I_{2S}A^{6OH}{}_{NAc}$	96.8 56.6 72.9 80.6 74.2 62.9	102.3 76.6 67.1 74.1 70.6
		5.14 4.03 3.79 3.76 3.91 3.87-3.92	5.26 4.37 4.28 4.07 4.91
**2-OH/6-OH**	$I_{2OH}A^{6OH}{}_{NS}$	98.2 60.5 72.5 80.2 73.5 62.4	104.3 72.2 71.5 77.8 72.2
		5.39 3.26 3.67 3.72 3.87 3.84-3.88	4.95 3.74 4.11 4.08 4.77
**2-OH/6-OH/N-Ac**	$I_{2OH}A^{6OH}{}_{NAc}$	97.1 56.2 72.3 79.6 73.7 62.3	104.3 72.5 72.2 77.3 72.6
		5.18 3.97 3.76 3.74 3.89 3.85-3.88	4.92 3.69 3.89 4.07 4.73
**Oversulfated (OS)**	$I_{2S,3S}A^{6S}{}_{3S,NS}$	99.6 59.3 82.9 76.8 72.1 68.7	100.8 73.6 72.9 73..3 69.8
		5.32 3.50 4.48 4.04 4.05 4.27-4.41	5.32 4.55 4.72 4.39 5.05

Table S1. ^{13}C NMR chemical shifts values (/ppm down field of TSP) of predominant repeating sequences in chemically-modified heparin derivatives. A-1 to A-6 and I-1 to I-5 represent positions around the rings of glucosamine (aminosugar): denoted A, or iduronate: denoted I. For the shorthand notations of polysaccharides and disaccharide repeats (columns on left), **2S**, **2OH**, **6S**, **6OH**, **NS** and **NAc** represent: sulfate (**S**), hydroxyl (**OH**) and acetyl (**Ac**) substitutions at positions 2- of iduronate, 6- of glucosamine or N- of glucosamine. Signals from the carbonyl group of iduronate and acetyl CH_3 groups of *N*-acetylated glucosamine derivatives are not shown. ^{13}C spectra were recorded using 150 mg of polysaccharides in D_2O (0.8 mL) at 40 °C on a 400 MHz instrument and are in agreement with the assignments previously reported (1,2).

A. Robo1 Ig1-5

```
                  .........10 .........20 .........30 .........40 .........50
DROME_ROBO_1-5    PRIIEHPTDL VVKKNEPATL NCKVEGKPEP TIEWFKDGEP VSTNEK--KS
CHICK_ROBO1_1-5   PRIVEHPSDL IVSKGEPATL NCKAEGRPTP TIEWYKGGER VETDKDDPRS
Consistency       ***8***5** 8*4*4***** ***5**6*3* ****6*3**2 *4*553006*

                  .........60 .........70 .........80 .........90 .........100
DROME_ROBO_1-5    HRVQFKDGAL FFYRTMQG-K KEQDGGEYWC VAKNRVGQAV SRHASLQIAV
CHICK_ROBO1_1-5   HRMLLPSGSL FFLRIVHGRK SRPDEGVYVC VARNYLGEAV SHNASLEVAI
Consistency       **52434*6* **3*353*0* 443*2*2*0* **6*26*6** *34***68*8

                  .........110.........120.........130.........140.........150
DROME_ROBO_1-5    LRDDFRVEPK DTRVAKGETA LLECGPPKGI PEPTLIWIKD GVPLDDLKAM
CHICK_ROBO1_1-5   LRDDFRQNPS DVMVAVGEPA VMECQPPRGH PEPTISWKKD GTPLDD----
Consistency       ******24*4 *43**2**3* 67**2**6*1 ****72*1** *4****0000

                  .........160.........170.........180.........190.........200
DROME_ROBO_1-5    SFGASSRVRI VDGGNLLISN VEPIDEGNYK CIAQNLVG-- ------VQV-
CHICK_ROBO1_1-5   ----KDERIT IRGGKLMITY TRKNDAGKYV CVGTNMVGER ESEVAELTVL
Consistency       0000444113 82**4*7*51 4431*3*4*2 *843*7**00 00000063*0

                  .........210.........220.........230.........240.........250
DROME_ROBO_1-5    -KPYFMKEPK DQVMLYGQTA TFHCSVGGDP PPKVLWKKEE GNIPVSRARI
CHICK_ROBO1_1-5   ERPSFVKRPS NLAVTVDDSA EFKCEARGDP VPTVRWRKDD GELPKARYEI
Consistency       06*2*5*4*4 525533345* 3*2*452*** 2*3*2*6*66 *47*26*24*

                  .........260.........270.........280.........290.........300
DROME_ROBO_1-5    LHDEKSLEIS NITPTDEGTY VCEAHNNVGQ ISARASLIVH APPNFTKRPS
CHICK_ROBO1_1-5   R-DDHTLKIR KVMAGDMGSY TCVAENMVGK AEASATLTVQ EPPQFVVKPR
Consistency       20*625*5*3 48332*2*5* 4*2*3*2**5 34*3*5*3*3 3**4*426*3

                  .........310.........320.........330.........340.........350
DROME_ROBO_1-5    NKKVGLNGVV QLPCMASGNP PPSVFWTKEG VSTLMF---P NSSHGRQYVA
CHICK_ROBO1_1-5   DQVAALGRTV TFQCEATGNP QPAIFWRREG SQNLLFSYQP PQSSSRFSVS
Consistency       55254*424* 343*2*5*** 3*68**36** 244*7*000* 14*34*12*6

                  .........360.........370.........380.........390.........400
DROME_ROBO_1-5    ADGTLQITDV RQEDEGYYVC SAFSVVDSST VRVFLQVSSV -DERPPPIIQ
CHICK_ROBO1_1-5   QTGDLTITNV QRSDVGYYIC QTLNVAGSII TKAYLEVTDV IADRPPPVIR
Consistency       33*3*3**5* 554*2***8* 4445*53*23 4656*6*54* 026****8*5

                  .........410.........420.........430.........440.........450
DROME_ROBO_1-5    IGPANQTLPK GSVATLPCRA TGNPSPRIKW FHDGHAVQ-A GNRYSIIQGS
CHICK_ROBO1_1-5   QGPVNQTVAV DGTLVLNCVA TGTLTPTILW KKDGTLISTQ DSRIKQLETG
Consistency       1**5***632 34434*1*1* **415*3*2* 12**138403 35*3417624

                  .........460.........470.........480...
DROME_ROBO_1-5    SLRVDDLQLS DSGTYTCTAS GERGETSWAA TLT
CHICK_ROBO1_1-5   ALQIRYAKLG DTGRYTCVAS TPSGEATWSA YIE
Consistency       6*582035*4 *5*3***4** 233**45*6* 273
```

Unconserved 0 1 2 3 4 5 6 7 8 9 10 Conserved

B. Robo2 Ig1-5

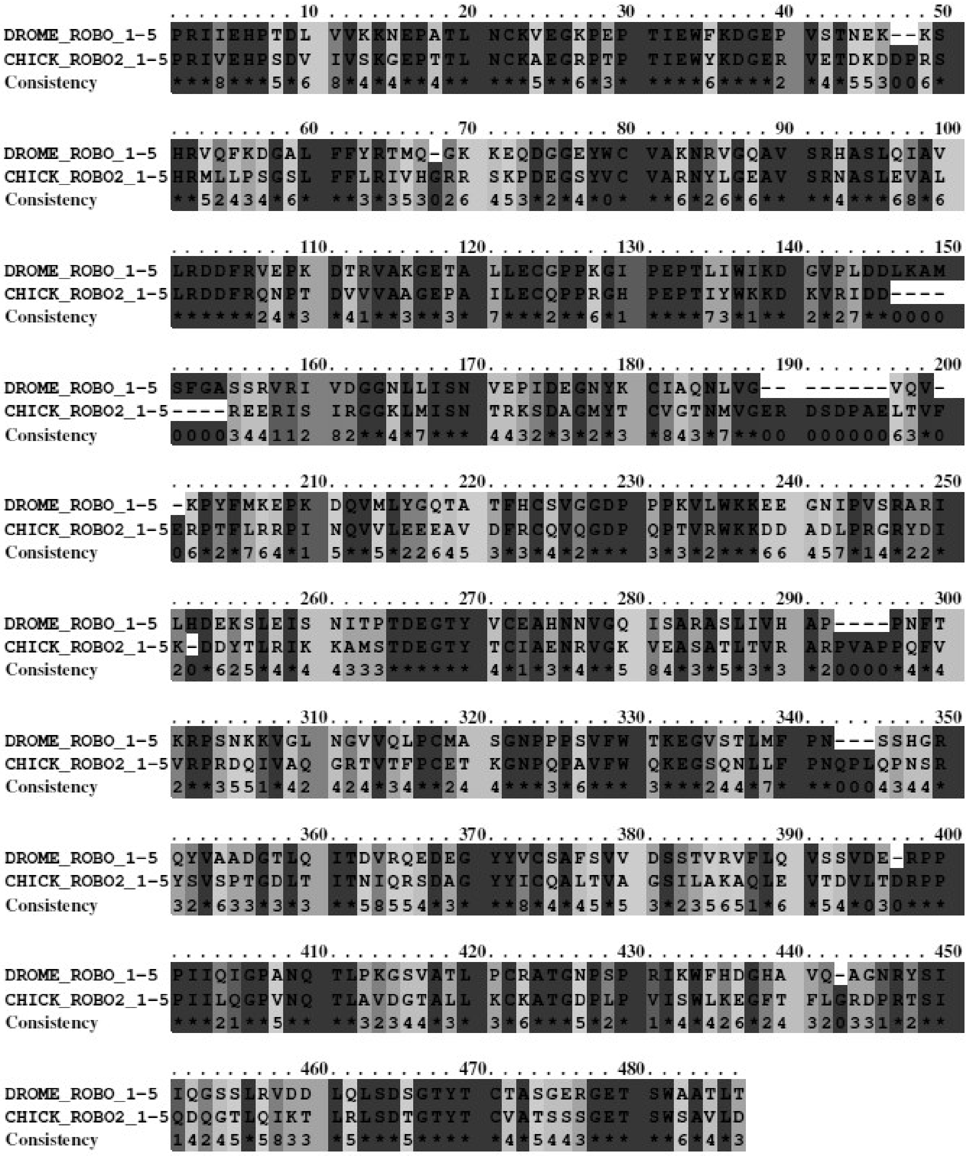

Figure S1. Amino acid sequence alignment. Amino acid sequences for **(A)** *Drosophila* (Drome) Robo and cRobo1 Ig1-5 and **(B)** *Drosophila* (Drome) Robo and cRobo2 Ig1-5 were compared using PRALINE. The conservation scoring scheme runs from 0 for the least conserved up to 10 for the most conserved.

A. dSlit D1-4 binding

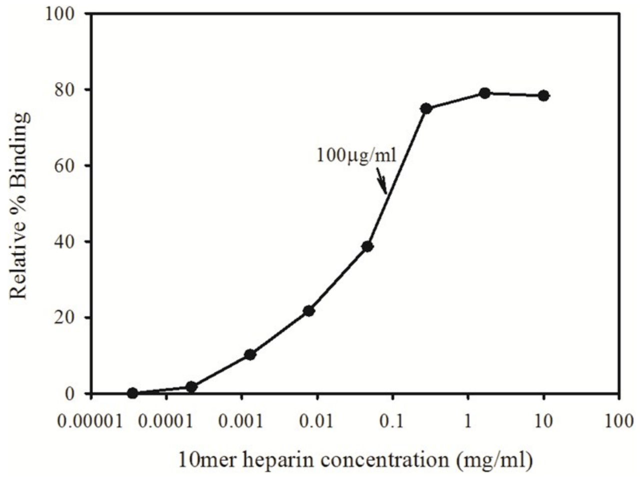

B. Robo Ig1-5-Fc binding

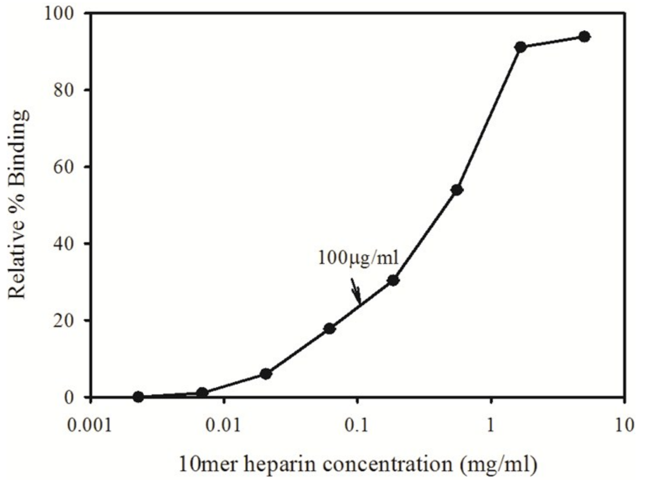

Figure S2. Binding of ~ 10-mer heparin SEC saccharide fraction to dSlit D1-4 and dRobo Ig1-5-Fc. The ability of varying concentrations of a SEC fraction containing ~10-mer heparin saccharides to bind to **(A)** his_6-cmyc dSlit D1-4 and **(B)** dRobo Ig1-5-Fc was determined using a competition ELISA. % binding values represent means of triplicate wells containing competitor relative to means of triplicate wells lacking competitor and error bars represent the % combined standard deviation calculated as described in Experimental Procedures. Data is representative of four separate experiments. Arrows represent the concentration of saccharide chromatographic fractions used in screening experiments.

A. dSlit D1-4

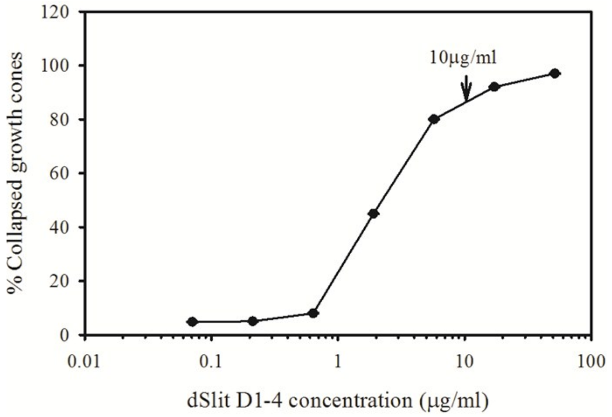

B. Heparin

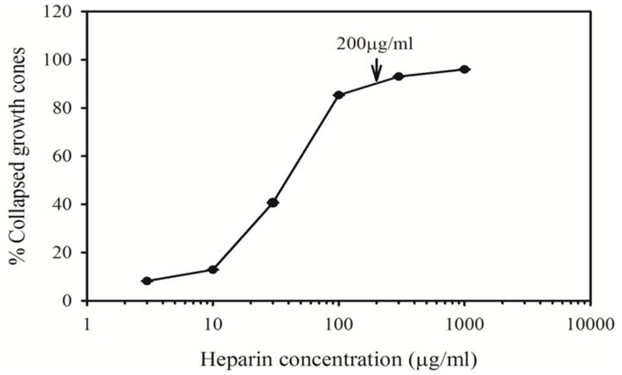

A. ~10mer PMH SEC fraction

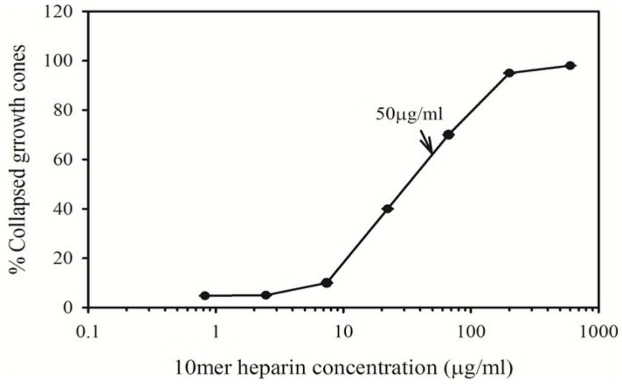

Figure S3. Effect of protein and GAG concentration on the cellular activity of dSlit D1-4. Slices of chick retina were treated *ex vivo* with **(A)** varying concentrations of his_6-cmyc-dSlit D1-4 or heparinases I-III followed by 10 µg/ml his_6-cmyc-dSlit D1-4 in the presence of varying concentrations of **(B)** heparin or **(C)** ~10mer heparin SEC fraction. Collapsed and uncollapsed growth cones were counted in blind conditions across several pieces of retina for ~100 growth cones and the % of collapsed growth cones calculated. Values shown are the mean % of collapsed growth cones calculated from three groups of retinal pieces and error bars represent the standard deviation for % values. Data are representative of two separate experiments. Arrows represent the concentrations of dSlit D1-4, polysaccharide variants and saccharide chromatographic fractions used in screening experiments.

REFERENCES

1. Yates, E. A., Santini, F., Guerrini, M., Naggi, A., Torri, G., and Casu, B. (1996) 1H and 13C NMR spectral assignments of the major sequences of twelve systematically modified heparin derivatives. *Carbohydr Res* **294**, 15-27
2. Yates, E. A., Santini, F., De Cristofano, B., Payre, N., Cosentino, C., Guerrini, M., Naggi, A., Torri, G., and Hricovini, M. (2000) Effect of substitution pattern on 1H, 13C NMR chemical shifts and 1J(CH) coupling constants in heparin derivatives. *Carbohydr Res* **329**, 239-247
